# A cerebral clue in the stomach: Brain lesion leads to discovery of hidden gastric cancer

**DOI:** 10.1016/j.ijscr.2024.110788

**Published:** 2024-12-25

**Authors:** Zahra Bahrami Samani, Mohammadreza Elhaie, Nadia Najafizade

**Affiliations:** a Department of Radio Oncology, School of Medicine Cancer Prevention Research Center Seyyed Al-Shohada Hospital Isfahan University of Medical Sciences, Iran; b Department of Medical Physics, School of Medicine, Isfahan University of Medical Sciences, Iran

**Keywords:** Gastric neoplasms, Brain neoplasms, Neoplasm metastasis, Neuroimaging, Adenocarcinoma

## Abstract

**Introduction and importance:**

Brain metastases from gastric adenocarcinoma are exceptionally rare, comprising only 0.1–0.16 % of all brain metastases. These cases present unique diagnostic challenges, particularly when neurological manifestations precede gastrointestinal symptoms. Understanding such atypical presentations is crucial for timely diagnosis and appropriate management.

**Case presentation:**

An 82-year-old male presented with non-specific neurological symptoms including dizziness, fever, and seizures, without any reported symptoms of primary gastric involvement. Initial neuroimaging revealed a left frontal brain lesion, which upon biopsy was confirmed to be an undifferentiated carcinoma. Subsequent positron emission tomography-computed tomography identified a primary gastric tumor with evidence of systemic metastatic spread.

**Clinical discussion:**

The case highlights the diagnostic complexity of brain metastases from gastric cancer, particularly when presenting without primary site symptoms. A comprehensive multi-modality diagnostic approach, including neuroimaging, histopathological analysis, and molecular studies, was essential for establishing the definitive diagnosis. Treatment involved a coordinated multi-disciplinary strategy combining systemic chemotherapy (capecitabine and oxaliplatin) with targeted brain radiation therapy.

**Conclusion:**

This case emphasizes the importance of maintaining a broad differential diagnosis and considering rare metastatic patterns, even in clinically atypical scenarios lacking primary tumor symptoms. It also underscores the value of comprehensive diagnostic evaluation and multi-disciplinary collaboration in managing such complex oncological cases.

## Highlights

• Rare case of gastric cancer presenting with brain metastasis (0.1–0.16 %)
• Unique presentation with neurological symptoms without GI manifestations
• Importance of comprehensive diagnostic approach
• Value of multi-disciplinary management

## 1 Introduction

Gastric cancer is a widespread malignancy worldwide, with high incidence and mortality rates globally [1,2]. While gastric adenocarcinoma commonly metastasizes to the liver, peritoneum, and other intra-abdominal organs, metastasis to the brain is exceedingly rare, constituting only 0.1–0.16 % of all brain metastases [3,4]. When gastric cancer metastasizes to the brain, it often poses significant diagnostic challenges due to the atypical clinical presentation and lack of pathognomonic symptoms [5]. Furthermore, a thorough understanding of the clinical characteristics and prognosis associated with gastric cancer brain metastasis remains limited due to the paucity of well-documented cases [6]. This case report aims to contribute to the scant literature on this uncommon pattern of metastatic spread. It documents the clinical progression and multidisciplinary diagnostic and treatment approach undertaken for an elderly male patient who presented with new-onset neurological deficits but no signs of primary gastric involvement. Through detailed radiographic investigation and histopathological analysis, the metastasis was conclusively determined to originate from a primary gastric adenocarcinoma. This case highlights the importance of considering rare metastatic variants, even in the absence of primary symptoms, to avoid diagnostic delays and enable individualized management strategies. It also reiterates the need for collaborative research efforts to enhance clinical insights into this poorly characterized phenomenon.

## 2 Case presentation

**Fig. 1:**
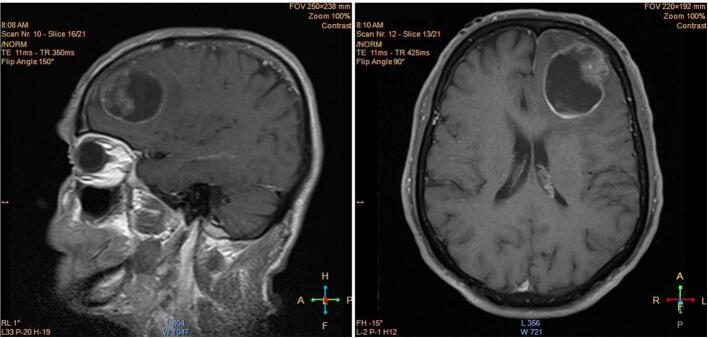
A lesion measuring 45 mm in diameter was identified within the intra-axial region of the left frontal lobe, characterized by the presence of surrounding edema and a notable mass effect. This lesion exhibited a significant cystic component located posteriorly, alongside an anterior component that appeared ill-defined.

**Fig. 2:**
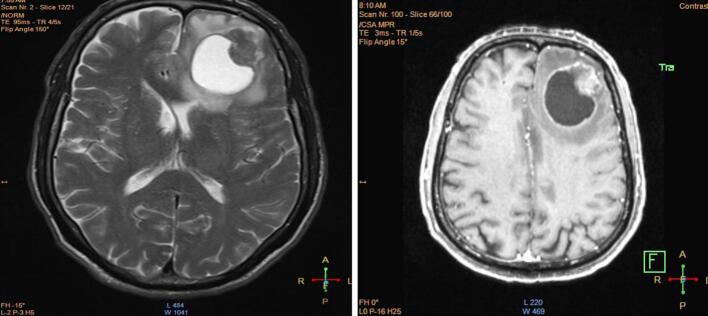
Magnetic resonance images of the brain demonstrating differences in T1- and T2-weighted contrast. Left image (T2-weighted): This axial slice was acquired with a turbo spin echo sequence using an echo time (TE) of 95 ms, repetition time (TR) of 4/5 s, and flip angle of 150°. T2-weighted images produce high signal intensity from fluid-containing structures such as edema due to the longer TE, making them optimal for visualizing pathology involving increased water content. Right image (T1-weighted post-gadolinium contrast): This coronal slice was obtained following intravenous administration of a gadolinium-based contrast agent using a spin echo sequence with a TE of 3 ms, TR of 1/5 s, and flip angle of 15°. In T1-weighted images, fat and tissues such as gray matter appear bright. Regions of blood-brain barrier disruption, as occurs with tumors, infections, or areas of inflammation, enhance following contrast administration due to leakage of gadolinium chelate into the extracellular space. This facilitates identification and characterization of lesion morphology and extent.

**Fig. 3:**
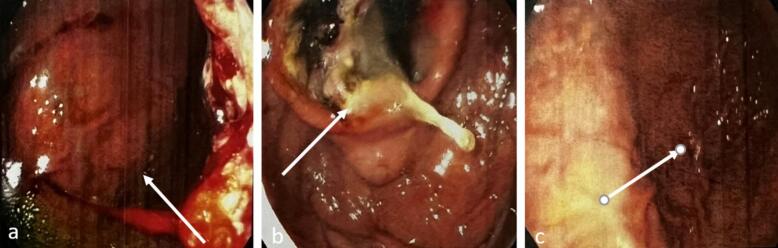
Endoscopic findings of a circumferential mass lesion located in the cardia and fundus, with biopsy performed. (a) Cardia: A mass lesion is observed with surface irregularities and possible ulceration, indicated by the arrow. (b) Fundus: The lesion extends into the fundus, showing a prominent nodular mass with surrounding inflammation, marked by the arrow. (c) Body: The lesion's progression into the body is evident, characterized by diffuse thickening and irregular mucosa, as shown by the arrow.

**Fig. 4:**
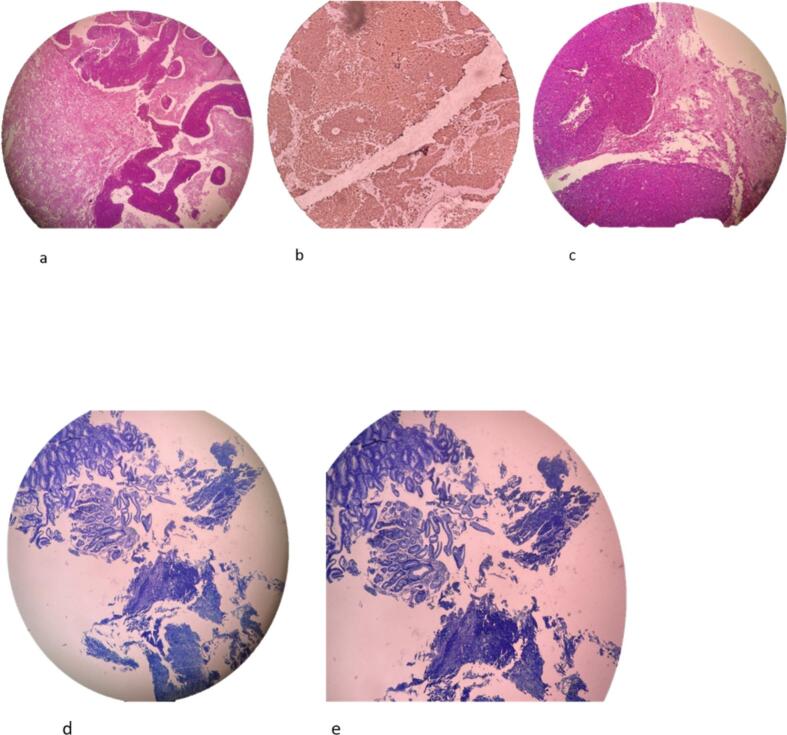
This light micrograph depicts a histological section of rodent brain tissue. Surprisingly, morphological features resembling gastrointestinal epithelium and glands are discernible embedded within the cerebral parenchyma (panels a–c). Additionally, photomicrographs d–e show sections of stomach tissue from the same rodent. Evidence of carcinomatosis can be observed, including malignant cellular infiltration and morphological changes indicative of neoplastic processes within the gastric mucosa.

An 82-year-old Iranian male presented to our medical institution's emergency department on July 9, 2023 complaining of dizziness, fever, and new-onset convulsions. His past medical history was non-contributory, with no prior diagnosis of gastrointestinal disease. The patient had no significant past medical or surgical history reported. The patient had fever but no night sweats or weight loss. No prior history of gastrointestinal disease or cancer. No relevant family history of note was reported. Upon initial evaluation, physical examination was notable only for intermittent myoclonic jerks and generalized tonic-clonic seizures. Comprehensive laboratory investigation failed to uncover an underlying cause, as standard blood work and biochemical assays yielded normal results and thus did not explain his neurological deficits. Laboratory investigations were conducted to discern potential causes underlying this gentleman's novel neurological manifestations and complaints of pyrexia, including febrile illness and inflammatory or infectious etiologies. Comprehensive bloodwork encompassed a complete blood count, which revealed a hemoglobin concentration of 13.5 g/dL (reference range: 13.5–17.5 g/dL), white blood cell count of 6.0 × 109/L (reference range: 4.0–11.0 × 109/L), and platelet count of 180 × 109/L (reference range: 150–400 × 109/L), all within normal limits. Tests of hepatic and renal synthetic function, including liver function tests, kidney function tests and serum electrolyte levels, were likewise within reference ranges. Inflammatory markers assessed comprised an erythrocyte sedimentation rate of 10 mm/h (reference range: 0–15 mm/h) and C-reactive protein level < 5 mg/L (reference range: <5 mg/L), denoting no overt evidence of systemic inflammation. Hence, standard laboratory examination failed to detect an underlying precipitant to account for his presentation. At this time, a GI origin was not suspected. The patient provided written informed consent for the publication of this case report and accompanying images. Moreover, the work has been reported in line with the SCARE criteria [7]. All efforts have been made to protect the patient's privacy and anonymity. Neuroimaging was conducted using a 1.5 T MRI scanner, which revealed an intra-axial lesion in the left frontal lobe. The MRI report detailed a lesion measuring 45 mm in diameter, characterized by surrounding edema and significant mass effect, with a notable cystic component. The MRI sequences included T2-weighted images highlighting fluid-containing structures and T1-weighted images post‑gadolinium contrast to assess blood-brain barrier disruption. Due to the patient's clinical presentation and the nature of the lesions observed, Diffusion Weighted Imaging (DWI) and Apparent Diffusion Coefficient (ADC) mapping were not performed as they were deemed unnecessary for the initial diagnosis of the brain lesion. Additionally, MR spectroscopy was not utilized because the primary concern was to establish the presence of metastatic disease rather than assess metabolic characteristics at this stage. The subsequent biopsy confirmed metastatic undifferentiated carcinoma originating from an undiagnosed gastric adenocarcinoma, as indicated by a PET-CT scan showing hypermetabolic gastric wall thickening. Brain computed tomography (CT) followed by MRI revealed an intra-axial lesion within the left frontal lobe. Subsequent histopathological analysis of lesional biopsy specimen via open craniotomy confirmed the presence of metastatic undifferentiated carcinoma. Immunohistochemically staining of biopsy samples was positive for pan-cytokeratin and negative for BCL2 and P63. Positron emission tomography-computed tomography (PET-CT) scan identified hyper metabolic gastric wall thickening, strongly indicating a primary gastric malignancy as the likely source of the cerebral metastasis. Collectively, the multimodal neuroimaging, histopathologic, and metabolic imaging findings established the diagnosis of brain metastasis secondary to undiagnosed gastric adenocarcinoma in this elderly male patient. This case underscores the importance of considering unusual patterns of metastatic spread even without corresponding primary symptoms, to facilitate precise diagnosis and management in complex oncologic cases. Upon initial physical examination, vital signs were stable yet notable neurological signs were observed. The patient experienced intermittent myoclonic jerks as well as episodes of generalized tonic-clonic seizures. Comprehensive laboratory workup including standard hematologic and biochemical analysis panels were undertaken to investigate potential etiologies. Laboratory results returned within normal limits and thus did not provide insight regarding the neurological presentation or complaints of dizziness and fever. Diagnostic Assessment Given the nonspecific clinical manifestations, further evaluation was pursued to elucidate the underlying pathology. Neuroimaging via non-contrast brain CT was performed followed by MRI with and without contrast administration. MRI revealed an intra-axial lesion within the left frontal lobe (Fig. 1, Fig. 2). Tissue biopsy of the lesional mass was then obtained during an open craniotomy procedure. Histopathological analysis of the biopsy specimen identified metastatic carcinoma (Fig. 3, Fig. 4). PET-CT scanning demonstrated hyper metabolic activity localized primarily to the gastric region, strongly implicating an occult gastric primary malignancy as the source of the cerebral metastasis. The PET-CT findings indicated multiple lymph node involvement, specifically in the perigastric and celiac regions, with enlarged lymph nodes noted, further supporting the diagnosis of systemic metastatic spread from the primary gastric lesion. Given the advanced stage with widespread metastases, curative options were limited. Palliative chemotherapy with the XELOX regimen (capecitabine 1000 mg/m^2^ twice daily, days 1–14 and oxaliplatin 130 mg/m^2^ on day 1, every 3 weeks) was initiated as first-line treatment. Concurrently, focal radiation therapy (total 24 Gy in three fractions) targeting the cerebral lesion was administered to alleviate neurological symptoms. The patient showed initial improvement in neurological symptoms, with treatment being generally well-tolerated. Close monitoring of treatment response and surveillance was maintained through regular follow-up.

Histopathological examination of the biopsy specimen retrieved from the left frontal brain lesion, combined with findings on integrated PET-CT, established the definitive diagnosis. Immunohistochemically analysis of the biopsy confirmed metastatic undifferentiated carcinoma. Marked hyper metabolic activity isolated to the gastric wall on PET-CT imaging substantiated the primary origin as gastric adenocarcinoma. This rare instance of brain metastasis absent concurrent gastrointestinal symptoms posed diagnostic challenges. The tumor cells showed positive staining for pan-cytokeratin (AE1/AE3), CK7, and CDX2, while being negative for CK20, TTF-1, and GATA3. This IHC pattern, combined with the morphological features of signet ring cells and poorly cohesive growth pattern, is highly specific for gastric adenocarcinoma. Treatment and Outcome: Given the advanced stage with widespread metastases, curative options were limited. Palliative chemotherapy with the XELUX regimen was initiated as first-line treatment of the gastric primary. Concurrently, focal radiation therapy targeting the cerebral lesion was administered in three fractions to alleviate neurological symptoms. Close monitoring of treatment response and surveillance for new signs/symptoms was instituted.

## 3 Discussion

Metastasis of gastric carcinoma to the brain is exceedingly uncommon, with sparse case documentation [6]. The atypical clinical presentation lacking abdominal signs further complicated diagnosis. This case highlights the importance of considering unusual metastatic patterns to facilitate timely diagnosis and guide multidisciplinary management, even without accompanying primary symptoms [8,9]. The exceedingly rare occurrence of brain metastasis from gastric adenocarcinoma, representing merely 0.1–0.16 % of all brain metastases, presents unique diagnostic challenges that merit careful consideration. While gastric cancer predominantly exhibits a predilection for hepatic and peritoneal spread, the underlying mechanisms facilitating cerebral metastasis—including hematogenous dissemination via the vertebral venous system and blood-brain barrier disruption—remain incompletely understood. This case is particularly noteworthy for its presentation with isolated neurological symptoms (dizziness, fever, and seizures) in the complete absence of typical gastric manifestations such as dysphagia, early satiety, weight loss, or epigastric pain. The successful diagnosis resulted from a systematic multi-modal approach incorporating advanced neuroimaging, histopathological analysis, immunohistochemical studies, and metabolic imaging via PET-CT, ultimately enabling the identification of the primary gastric malignancy. The management strategy exemplified the necessity of coordinated multi-disciplinary care, combining systemic chemotherapy (XELOX regimen) with targeted radiation therapy for brain metastasis. While the prognosis for gastric cancer with brain metastasis generally remains poor, with survival typically measured in months, this case underscores the critical importance of maintaining a broad differential diagnosis, implementing comprehensive diagnostic strategies, and initiating prompt intervention, even in the setting of atypical presentations lacking primary site symptoms.

## 4 Conclusion

This report elucidates the rarity of gastric Cancer spread to the brain and difficulties in diagnosis absent gastrointestinal symptoms. It also underscores the value of multimodal therapy and underscores the need for coordinated specialty care. Further studies can improve understanding of this distinctive metastatic pattern and support personalized approaches in advanced disease.

## Author contribution

Zahra Bahrami Samani: Conceptualization, clinical management of the case, investigation, data collection, writing - original draft preparation, supervision, and final manuscript review and editing.

Mohammadreza Elhaie: Data analysis, technical support, interpretation of imaging studies, writing - review and editing, visualization of figures and imaging data.

Nadia Najafizade: Clinical management of the case, methodology, validation, writing - review and editing, project administration, and clinical supervision.

All authors have read and agreed to the published version of the manuscript.

## Consent

Written informed consent was obtained from the patient for publication of this case report and accompanying images. A copy of the written consent is available for review by the Editor-in-Chief of this journal. All efforts have been made to protect patient privacy and anonymity.

## Ethical approval

As per our institutional policy, ethical approval is not required for case reports where informed consent has been obtained.

## Guarantor

Nadia Najafizade: Clinical management of the case, methodology, validation, writing - review and editing, project administration, and clinical supervision.

## Research registration number

Not applicable.

## Funding

No funding was received for the preparation and publication of this case report.

## Conflict of interest statement

The authors declare no conflicts of interest regarding the publication of this case report.
